# Temperature of Yellow Fever

**Published:** 1874-04

**Authors:** 


TEMPERATURE IN YELLOW FEVER.—By Joseph Jones, M.D., Professor of Chemistry and Clinical Medicine in the Medical Department of the University of Louisiana, Visiting Physician of Charity Hospital, New Orleans, Louisiana: Pamphlet, pp. 6.

On Sarcomatous Growths of the Uterus. By Dr. William F. Jenks, Surgeon to the State Hospital for Women. Philadelphia: 1874. Pamphlet, pp. 15.

---

Relations of Colorado to Pulmonary Consumption. By Thomas E. Massey, A.M., M.D. Denver: 1874. Pamphlet, pp. 26.

---

Yellow Fever Epidemic of Memphis, Tennessee, 1873.—As observed by Y. R. LeMonnier, M.D., Visiting Surgeon Charity Hospital, New Orleans: Pamphlet, pp. 48.

---

Changes in Temperature and Pulse in Yellow Fever. By Joseph Jones, M.D., Professor, etc. Pamphlet, pp. 19.

